# Use of digital media for family planning information by women and their social networks in Kenya: A qualitative study in peri-urban Nairobi

**DOI:** 10.3389/fsoc.2022.886548

**Published:** 2022-08-04

**Authors:** Anja Zinke-Allmang, Rahma Hassan, Amiya Bhatia, Krittika Gorur, Amy Shipow, Concilia Ogolla, Sarah Shirley, Kees Keizer, Beniamino Cislaghi

**Affiliations:** ^1^Department of Global Health and Development, London School of Hygiene and Tropical Medicine, London, United Kingdom; ^2^Institute for Development Studies, University of Nairobi, Nairobi, Kenya; ^3^Busara Center for Behavioral Economics, Nairobi, Kenya; ^4^Harvard College, Harvard University, Cambridge, MA, United States; ^5^Faculty of Behavioural and Social Sciences, University of Groningen, Groningen, Netherlands

**Keywords:** family planning, digital media, Kenya, gender, social norms

## Abstract

Access to information about family planning (FP) continues to have financial, physical and social barriers among young women living in Kenya. This paper draws on social norms theory to explore how young women and their social networks access FP information on digital media (e.g., WhatsApp, websites). Qualitative phone interviews were conducted with 40 participants – young women, their partners and key influencers – in seven peri-urban wards in Nairobi, Kenya. Data were analyzed using thematic analysis. Findings suggested that young women, their partners and key influencers predominately accessed FP information online through their informal networks, but identified healthcare workers as the most trusted sources of FP information. In digital spaces, participants described being more comfortable sharing FP information as digital spaces allowed for greater privacy and reduced stigma to talk about FP openly. Our findings highlight the importance of digital media in disseminating FP information among young women and their networks, the differences in norms governing the acceptability to talk about FP online vs. in-person and the significance of targeting misinformation about FP in digital media spaces.

## Introduction

Health promotion strategies that use digital media to disseminate information have expanded rapidly in recent years (Volkmer, 2021). Where there is internet and smart phone access, digital media–an umbrella term that describes any methods of digital communication including texts, websites, social media apps, etc.–provides opportunities to remove barriers in accessing accurate sexual and reproductive health information and provides social networks online resources to share family planning (FP) information (Ochako et al., 2015; United Nations Population Fund, 2017). Previous interventions to improve access to FP information found that targeting the channels in which social networks communicate has been key to address misinformation about FP (Nazzar et al., 1995; Colleran and Mace, 2015; Boydell et al., 2020). Studies in Kenya point to the role of health care providers (Alege et al., 2016) and key influencers, such as close family and friends, as trusted sources of FP information (Hassan et al., 2021a)^1^. As more young people have access to the internet, digital media and technology in Kenya, healthcare providers have turned to digital media to develop new ways of sharing credible FP information with multiple groups, with an emphasis on young people (Zhou et al., 2018).

While FP use in sub–Saharan Africa has remained low, Kenya has had a 12.5% increase in FP use between 2008 (45.5%) and 2014 (58%) (Kenya National Bureau Of Statistics, 2015). Although knowledge on FP in Kenya is high, with 98% of women and 99% of men knowing of any modern FP method in 2014 (Kenya National Bureau Of Statistics, 2015), studies have pointed to problems of accessing FP, inadequate availability of preferred FP methods and lack of accessible FP information as key barriers (Montez, 2011; Engelbert Bain et al., 2021). Concerns on the low prevalence of FP uptake have stimulated both policy and program interventions aimed at enhancing FP access and information through digital media interventions (Welch et al., 2016; Yousef et al., 2021). Evidence shows that the provision of targeted, easily accessible, and accurate information about FP through multiple channels increased the use of modern contraceptives and influenced social norms on its utilization (Oluwasanu et al., 2019; Ahmed and Seid, 2020). For instance, a study in Ethiopia reported increased likelihood of women using FP among those who saw FP messages on television (Ahmed and Seid, 2020) and a cross sectional study in Kenya, Nigeria and Senegal found women who listened to FP messages on radio had higher rates of FP use (Okigbo et al., 2015). However, there is limited research on how women access FP information through digital media in low-income settings, how they would prefer to access FP information and the normative context within digital media that affect discussing and sharing FP information.

Engagement with digital FP information and women's FP decisions are influenced by the social norms related to FP (Costenbader et al., 2019) in their context (Simkhada et al., 2010; Wegs et al., 2016), which may deter women from accessing FP information both in-person and online. Group norms are shaped by the expressed attitudes of group members, which influence others attitudes to conform to group norms (Cislaghi and Shakya, 2018), a phenomenon which has been observed in both digital spaces and in-person groups (Levitan and Verhulst, 2016). Further, the gender composition of digital media spaces may influence women's FP decisions as gender norms are important factors that influence FP use (Cislaghi and Heise, 2020). The influence of digital platforms on FP conversations is crucial to understand as more conversations move online during COVID-19 and with increasing access to technology and internet.

The COVID-19 pandemic reduced access to FP services for women and drew further attention to how FP information can be accessed through digital media. With the shift of resources and attention to the pandemic, governments deprioritized FP funding, limiting accessibility to FP methods and information at health facilities (John et al., 2021; United Nations Population Fund, 2021). Research conducted by the United Nations Population Fund (UNFPA) predicts that more than 47 million women could lose access to contraception leading to 7 million unintended pregnancies as a result of the COVID-19 crisis (United Nations Population Fund, 2020). This gap in access to FP services disproportionally affects young people, who experience unique barriers to accessing FP methods and information (Prata et al., 2013; Engelbert Bain et al., 2021; Hassan et al., 2021a). In another study, we found that women in peri-urban Nairobi faced additional challenges accessing FP information and services during the pandemic and relied on digital media to reach their social networks to discuss FP and to seek information online (Hassan et al., 2021a).

Mobile phone ownership and digital media use has risen among young people since 2010 as access to the internet has increased in Kenya (Communications Authority Of Kenya, 2019). While the expansion of technology and adoption of mobile phones globally provide opportunities to reach more people and deliver health care services, new inequities have arisen as a result. Low-income areas with less access to technology, like smartphones, and internet connectivity have encountered new barriers to accessing FP information online (SIMElab, 2020). In informal settlements in Kenya, access to information is further constrained due to the poor infrastructure and internet connection in the area (Gichuna et al., 2020). Young women living in low-income peri-urban settings disproportionally experience barriers to accessing FP methods and information, which has been further entrenched by the COVID-19 pandemic (Hassan et al., 2021b).

There is limited research on preferred avenues of accessing FP information online among young women and their key influencers in low-resource settings, which is critical to address the challenges of unmet need of FP as well as removing barriers to FP information. Data for this study come from a larger study on the potential of online networks to initiate social norms change on offline networks, where the initial analyses of these data suggest that COVID-19 changed the normative influence of FP and limited access to women's social network and affected how they negotiated FP use within homes (Hassan et al., 2021a). Further, we found that women consult various key influencers in their social networks based on the information they need or the choices they are making in relation to FP (See footnote 1). To contribute to the literature on the opportunities and limits of using digital media to improve access to FP information, this paper draws on social norms theory to explore how young women living in peri-urban wards in Nairobi use and navigate digital media spaces to find FP information. To explore the normative influence of digital media on young women's attitudes and behaviors in accessing FP information online, we present why women prefer to use different sources of information and from whom they prefer to and do find information about FP on digital media.

## Methods

### Study design

Qualitative interviews were part of the formative phase of a mixed methods study to design and test a digital media intervention to examine how an online digital media intervention influences social norms around FP use among young women.

### Study sites and participants

We conducted 40 in-depth interviews in peri-urban wards within Nairobi, Kenya with women age 18–25 years, their partners and their key influencers to understand social norms supporting or condoning accessing information about FP and seeking FP services. Peri-urban wards were purposively selected based on whether they had health centers and FP services operational at the time of data collection so referrals to health services functioning during the COVID-19 pandemic could be provided to participants if requested.

All participants were sampled from a panel of 66,407 participants the Busara Center for Behavioral Economics recruited between 2014 and 2020. Due to safety considerations of remote data collection during COVID-19, women had to have their own (not shared) smartphone to participate in the study and were briefed to use a safe word if the interview were no longer private. Key influencers and partners were purposively selected based on similar sociodemographics to what women described during interviews, but were not related to the women who were interviewed due to safety concerns related to conducting interviews during the early stages of the COVID-19 pandemic. 41/89 women, 23/27 partners and 23/25 key influencers contacted were eligible to participate. Of these, 8 women, 11 partners, 7 key influencers declined to participate in the interviews due to work conflicts, illness and discomfort with the study topic. Our final sample included 16 women (W) between 18 and 25 years of age, 10 male partners (P) and 14 key influencers (KI), who women identified as people they would get advice about FP from, of whom 4 were men (M) and 10 were women (F).

The median age of among women interviewed was 23 years. Most women were unemployed (9/16) and had some secondary education or higher. More than half of the women interviewed were using contraception. Partners had a median age of 27.5 years, most of whom had secondary or university education (8/10) and were employed (7/10). Key influencers had a median age of 32 years, most of whom were partnered (11/14), had 3-4 children (6/14) and were employed (11/14).

### Data collection

We developed a semi-structured qualitative interview guide that consisted of three sections: a vignette to explore social norms and attitudes around FP and identify people in women's social networks who supported and opposed FP; questions about women and their key influencers use of digital media to access and share information on FP; and questions about how COVID-19 affected access to and use of FP. This instrument was piloted with participants with similar characteristics prior to data collection. This paper focuses on participant's use of digital media platforms to access FP information through formal and informal networks online, social norms and sanctions of accessing and using FP, and the effects of COVID-19. We define formal networks as community stakeholders or systems that provide FP methods or information (eg, healthcare workers) and informal networks as individuals that have personal relationships with participants and do not work formally in the field of healthcare or FP (eg, friend, partner). Women, partners and their key influencers were asked to reflect on their own experiences and those of other women in their community.

Data collectors were recruited who were familiar with the local context and had experience with qualitative interviewing. Prior to data collection, researchers completed a 3 day training for this study. Data collection was phone-based and took place in November 2020 during the COVID-19 pandemic. Researchers assessed eligibility, explained the study design and scheduled a phone interview time by phone and information about the study was also sent *via* WhatsApp. Consent was audio recorded and was iteratively checked-in throughout interviews through safe words and check-ins about privacy and comfort. Interviews among women were conducted by female interviewers and male participants were interviewed by either male or female researchers. Interviews were conducted in either English or Swahili, depending on the participant's preference. Participants were sent phone credit and a list of local resources and health facilities following the interview. A Busara researcher who had not conducted the interview translated and transcribed the interview and a random selection of transcripts were checked for quality. The research team held daily debriefs with the researchers to discuss issues and themes arising in the interviews.

### Data analysis

We developed a codebook using deductive and inductive methods (Azungah, 2018). A thematic analyses was conducted. We first coded transcripts by both using the codes in the codebook and remaining open to emerging new codes. Coders met regularly to discuss codes and themes arising from the data that were integrated in the codebook. To identify different ways young women and their social networks use digital media to seek and share FP information through their informal and formal networks, for this paper, we focused on themes that described 1) how women and their networks navigate digital media spaces to find and share FP information and 2) the norms and barriers experienced when accessing FP information through digital media.

### Ethical approval

This study received approval from the ethics committees in Strathmore University, Nairobi (ref SU-IERC0898/20) and the London School of Hygiene and Tropical Medicine (ref 22480).

## Results

Most women and their key influencers reported using digital media regularly, especially social media apps such as Facebook and WhatsApp. Participants described social media apps as “private”, “secure” and cost-effective ways of connecting with social networks in lieu of being able to connect in-person, especially during COVID-19 lockdowns. Social media channels provided avenues to access information about FP from various sources, where young women, their partners and key influencers found most of their information through casual online conversations or through social media posts about FP in both private and group conversations. First, we found that informal networks were common ways young women, their partners and key influencers shared and found FP information through friends and family over private messages. Second, participants trusted information from formal healthcare providers to provide accurate information about FP, which were mainly accessed through community groups on social media. Third, women, their partners and key influencers navigated digital media group spaces differently depending on the gender of group members, which influenced how comfortable they felt sharing information or experiences with others online.

### Using online media to access and discuss FP information online with informal networks

Participants described that digital media spaces offered a unique opportunity to access FP information online easily regardless of location, allowing for flexible access during working, care or other hours of duties: “*If you set up a WhatsApp group and share information, you can just log in and read them [messages with FP information] and spread them around when you have time*” (KI35_F). FP information was most commonly shared within social networks because it would be beneficial to inform friend's or family's FP choices in accessible ways, “*we can exchange ideas and information on family planning and benefit from each other*” (KI27_F). Some participants found the online discussions with individuals or small groups were useful spaces to access FP information through private or discrete channels: “*[On WhatsApp] I am the only one who can access [the message about FP information] unlike in Facebook where people read it especially if it is posted on the wall”* (P21). These conversations created a space where participants could discuss FP information to assess “*if [FP] is appropriate or not [to use]” (*KI29_F). Women also sought to validate whether the information they had received was accurate by “*asking if [other women] too had heard the same things [about FP methods]*” (W2). Communicating online with informal networks allowed for discreet ways to share FP information that otherwise might not be shared by speaking face to face, “*There are things I may want to share with you but I cannot in-person because I will feel shy but with WhatsApp or Messenger, one is very comfortable sharing*” (KI35_F). Digital media channels were useful tools for women to communicate with their informal networks individually or in small groups to receive and share FP information privately and securely, providing more opportunities for information on FP to be shared.

Despite appreciating how useful digital media were in accessing FP information, participants did not feel comfortable sharing FP information through digital media channels as they anticipated negative reactions about posting FP information from others online, “*I will be [comfortable] only if I have a positive response [talking to women online about FP]*” (P26). Several partners described feeling uncomfortable talking about FP online, since most FP methods are used by women and they lacked the lived experiences to talk about the methods, “*You know I am a guy, how do I start telling ladies about such*” (P19). Other men felt uncomfortable sharing or discussing FP information online with their male friends since starting such a conversation about FP is not common in-person, “*How can I start talking about family planning with my friends? So I rarely share*” (P26). In contrast, women found it more comfortable to speak to other women online, where online conversations offered new avenues of discussion about FP among women.

In addition to texting and WhatsApp groups, many participants accessed FP information through online groups created specifically for other social purposes, such as a women's community group or a sports group. Participants described recreational groups as spaces to access a variety of topics on health and FP, where one participant described that even groups that mostly meet in-person will share information through digital channels: “*We have a chama [group] for women and we communicate [about FP] via WhatsApp*” (KI33_F). Participants who are part of such groups felt more comfortable to share FP information, as they knew the others in the online group and knew that it was an effective way to share reliable FP information widely, “*When I get to share that [FP] information with them, they are more likely to share with other people*” (P23). These online groups provided a way to share accurate information on FP “*to create awareness”* (KI40_M) about the benefits of FP “*so as to inform those who fear to talk about this conversation*” (P20). While most participants connected with their informal social networks to access information on FP through social media channels, many participants preferred to receive FP information from credible sources such as healthcare providers.

### Accessing FP information online through formal healthcare sources

While the majority of participants still accessed information about FP through in-person health settings, such as hospitals or clinics, some described noticing and reading FP information indirectly through information advertised through posters or other printed media at healthcare settings which they trusted: “*In hospitals as we queue some social health workers advise us [about FP]. Also, the posters around hospitals and on roads [advise about FP]*” (W12). Participants would often use other health related visits at healthcare settings to access FP information, such as through “*the clinic, when taking the child”* (W10) or at regular visits: “*[I access FP information] from the hospitals when I visit every three months”* (KI28_F). Clinics were also identified as spaces that provide frequent in-person FP information sessions, where community members could be “*informed and given condoms to distribute and share in [the] area”* (KI35_F). Visiting clinics and hospitals in-person were useful not only for receiving credible information about family planning, but also for receiving the necessary family planning methods: “*I use pills and I ask questions from the doctor in the hospital in case I experience any side effects”* (W4). Healthcare centers and clinics provide clear spaces in which information on FP is reliable, a reputation of credibility which extends into posting information on online platforms.

Healthcare workers were perceived by participants as the most credible source of online FP information, “*[I] prefer people from health centers...not just any person [but] someone who has trained about family planning and is well knowledgeable”* (W7). Participants preferred to access information from healthcare providers online as they risked receiving misinformation about FP from other sources as, “*there are many myths [about FP] from people*” (KI36_F). Many participants identified examples of misinformation regarding FP messages online, in particular related to side effects of FP use: “*Most people think that using family planning especially if you haven't had children will have effects on you and that can make one fail to bear children*“ (KI27_F). However, most participants described that they would only avoid sharing information on FP if they deem it to be false or “*[if] it is a lie*” (P25). Other participants described difficulties in challenging misinformation about FP: “*They say such kinds of things and even if you go telling them about family planning, they already have a formed opinion that you cannot tell them anything*“ (KI35_F). Despite the misinformation that participants encountered both in-person and online from social networks, they also found that social networks gave advice to connect with healthcare workers for credible FP information,

“*The advice that I saw is that one shouldn't just use any family planning method that they come across instead they should go for the best that suits them and that you can find out which is the best by visiting a health expert for consultation and advice on family planning*.” (P21)

While healthcare providers were valued as credible sources to provide FP information, they were primarily reached through in-person interactions which became challenging during COVID-19 lockdowns, pushing participants to find information from sources within reach, “*I read [about FP] through social media since going to the hospital is not easy right now”* (KI34_M). Participants often sought out credible online counterparts to hospitals and clinics, such as clinical websites “*because there are professionals posting and writing”* (KI37_F). Another participant mentioned finding FP information through a youth Facebook group where nurses and doctors disseminated direct information about FP, which was accessible during the COVID-19 pandemic. However, many participants said that due to the financial impacts of COVID-19, they had more limited access to the internet: “*Where I worked I used to have access to a wifi-connection for free. Now I have to buy bundles and so it's very expensive sometimes*” (KI37_F). Despite finding in-person FP information delivered by healthcare providers or other FP organizations valuable, participants struggled to access this advice during the pandemic and more often sought FP information through medical websites or their informal networks.

### Navigating digital media spaces to share FP information

Although many participants were comfortable accessing FP information in online spaces since “*it's [FP information] all over, it's very normal*” (KI33_F), experiences using digital media to seek or share FP information varied based on the perceived membership of the social network or online forum. Most participants felt more comfortable speaking with women online than with men or strangers since they anticipated most other women were using or had used FP, “*I will be comfortable [speaking to women online about FP] because they all use it*” (KI37_F). Women found speaking with other women online the most comfortable, since this offered space for mutual learning about FP information and to learn about other women's experiences:

“*She is my fellow woman. It could be she has passed through the same things as I am. We could be having different experiences and different doctors. This means we could be sharing different experiences, and information which we have heard from our doctors. She could be having different information from me which I can exchange. I could also share with her something she doesn't know*.” (W4)

Many women said that it was important to inform other women about their FP options as a matter of improving the lives of women in their communities: “*Not speaking out [about FP information] will be leaving out the women with no knowledge. Society has to progress*” (W12). However, men felt less comfortable speaking with women online, because women are the predominant users of FP methods and have lived experiences: “*You can always share information with anyone, but then it's women who have more knowledge on family planning*” (P19). While men felt less comfortable talking to women about FP online, women were comfortable finding and sharing FP information with women they don't know online in similar ways they might share FP information among their personal social network.

Women and their key influencers were overall less comfortable speaking to men than women online since FP is mainly seen as a women's issue, “*Many [men] will not understand as it's a woman thing*” (KI37_F). Participants anticipated that men would have less experience using FP, “*family planning is mostly done by the women. Only few men agree to use family planning*” (KI29_F), and anticipated more negative responses about discussing FP from men online than from women or strangers: “*They [men online] would start throwing insults at me*” (P24). Women in particular felt uncomfortable speaking to men online because they did not feel safe to do, anticipating unwanted sexual advances online, “*Men are very hard to deal with because when you post something they will not take it seriously, maybe just a few of them will. Instead they will just want to meet up with you*” (W6). Others anticipated men reacting to FP information with negative attitudes and misconceptions toward FP that would make it difficult to interact with them online, “*They would not support me because they say women who use family planning are ‘not sweet”'* (W7), referring to the misconception that sexual intercourse with women who use FP is less enjoyable or pleasurable. Despite anticipating negative reactions and attitudes toward FP from men, women described the importance of still interacting with men to spread accurate information on FP to better the lives of other women and reduce violence due to misconceptions around FP:

“*Men are the main perpetrators against women in this community. If you don't teach men [about FP] they will always say the information we pass to women is toxic and it may bring domestic violence, so it's wise to teach both genders*.” (W12)

Some men described talking to other men online as a positive experience since they would be able to connect about various topics including FP, “*Men have courage, so we will ask each other deep questions*” (P22). Similar to women's experiences speaking to other women, men found this comfort speaking among other men online due to similar experiences, “*because we are the same gender and the experiences are the same*” (P20). A few participants said connecting with men online about FP is important “*so that they [men] do not leave the family planning issues to women alone*” (KI39_M).

Group conversations in digital media spaces also provided opportunities for conversations with strangers online. Anonymity in online group conversations also increases comfort to talk about FP online openly, “*These are people I do not know so I would be comfortable speaking to them online. I wouldn't want it to be people I know*” (P19). There was a certain protection in anonymity in speaking to strangers, where participants might change how they discuss FP information based on reception from others:

“*Online is okay because we don't meet, I can tell you anything because you can't meet me and we don't know each other or where I come from. I limit myself when we meet face-to-face as I could be considerate of how you feel or facial expression.”* (KI36_F)

Some participants described not being sure “where to start” a conversation about FP and were concerned that “*you can be liked or disliked at equal measures*” (P20) or judged based on the quality or accuracy of information posted since “*some [people online] just want to measure your intelligence*” (P22). However, some participants were still willing to connect with strangers online since “*they will give me any advice I might need on family planning*” (W9). Although there is concern that strangers online might react poorly to FP information being shared, participants were still keen to share information for the purpose of spreading good information about FP among others.

## Discussion

This qualitative study drew on data from 40 interviews with women, their partners and key influencers living in peri-urban Nairobi. All participants had access to a personal smartphone and we explored how young women and their social networks access and share FP information through digital media. We find that participants used digital media platforms to access FP information through both informal (e.g., WhatsApp groups with friends and family) and more formal (e.g., information posted on social media by a FP organization) sources online. There were important overlaps between in-person and online sharing of information and participants described visiting health providers in-person as a valuable way to seek advice on FP. Further we found evidence that participants specifically valued accessing credible FP information from gender homogenous online communities for a sense of safety to discuss and share FP information. Online groups with gender differences changed the normative context and shaped the level of online engagement to share FP information freely.

Digital media platforms, specifically social media platforms (e.g., WhatsApp, Facebook), are increasingly becoming a common way of communicating and provide flexible methods of accessing FP information at various stages throughout the FP use cycle. Although participants in our study had access to smartphones, they did not have reliable or consistent access to the internet due to financial strain during COVID-19. Young women and their networks were aware that others in their communities would not have such access to digital media and technology due to differences in incomes and financial responsibilities. However, for those who had some access to digital media during COVID-19, this meant that even in the context of large disruptions in in-person service delivery, people were not cut off from needed sexual and reproductive health information and even reach more people with accurate information on FP through digital media platforms. Our findings reveal that women and their social networks preferred to access information from healthcare providers online compared to information from their peers and found that this was an acceptable way of accessing trusted information when in-person services were not accessible during COVID-19.

Increasing the availability of FP information through multiple channels is critical to reach wider audiences that may not have access to in-person services and address unmet FP needs, which has been linked to lack of credible and trusted sources of FP information among women (Sedgh et al., 2016). Mass media programmes have been effective in reaching wide audiences through common channels such as TV or radio, but have been linked to missing key groups due to issues with when programming is aired and accessible (Volkmer, 2021). We found that digital platforms provide flexibility in how and when FP information is accessed, accommodating groups that are seeking trusted FP information, but face physical barriers to tune in to a program at a specific time or finding time or resources to get to a clinic. Our findings show that participants are interested in receiving and sharing FP information through digital platforms, which removes various known physical barriers to accessing FP information in sub-Saharan Africa (Bearinger et al., 2007). Our findings show young women and their networks were looking to share FP information within their online networks, which may move from offline to online spaces through informal conversations with friends and family. Although our findings did not uncover specifically what channels and digital media spaces participants accessed to actively find FP information, our findings did support other literature that young women and their networks trusted healthcare providers most to provide credible FP information.

Our findings also support other research that young women mainly find FP information through their social networks, which extended into digital spaces. We found that participants preferred to speak to their social networks about FP in-person, likely due to the possible sanctions and negative reactions from their community about accessing information on or using FP, but also discussed FP *via* WhatsApp. We also found that information received in-person was then shared digitally with others, typically through private individual or group conversations. Group conversations with informal social networks on social media platforms, such as WhatsApp and Facebook, were important spaces for young women to access FP information that normalized sharing FP information (Castle and Silva, 2019). Women in peri-urban areas navigate a complex normative context where speaking openly about FP can result in negative sanctions, such as gossip or bringing shame upon a woman or her family (Zinke-Allmang et al.). The value of secrecy in accessing FP information has been noted in other research (Mitchell et al., 2014; Brittain et al., 2018) (See footnote 1): in this context, digital platforms provide discrete spaces to share and validate FP information and where anonymity, privacy and secrecy are key aspects that promoted sharing FP information online (Cartwright et al., 2019). Participants identified online platforms as safe spaces because their identities were not known by others, a finding that is in line with other studies that highlight the value of anonymity on digital media platforms to avoid sanctions around accessing FP information (Mitchell et al., 2014). However, our findings reveal that participants' preferences in how to access FP information differed as some were comfortable connecting with their social ties openly while others preferred remaining anonymous in online groups.

Understanding the complex processes of cultural and gender dynamics that hinder access to information, especially in online settings, is critical to support women seeking to access FP methods and addressing unmet need in Kenya. While our findings reflect that women prefer to access and share FP information based on familiarity and social ties, others report that women's choice of health information is largely influenced by the trust they have in the source (Das and Sarkar, 2014) (See footnote 1). While mass media campaigns are the most common ways of disseminating FP information, they have been criticized for missing key groups and using digital platforms that are one-way in nature, such as TV or radio (Castle and Silva, 2019). In particular, our findings highlight the importance of targeting and engaging men and partners in FP discussions as they were not comfortable communicating online about FP despite valuing the dissemination of accurate FP information online, a similar pattern to other studies on men discussing FP in-person (Kabagenyi et al., 2014; Kriel et al., 2019). In contrast, women found community among other women online through common experiences using FP, enhancing access to FP information and safe avenues for sharing FP information. This discordance, between women's comfort in discussing FP online with other women and men's discomfort discussing FP experiences with other men, influences the dynamics between women and their partners when making decisions about FP use. This finding highlights the importance of including FP information in digital media spaces that are targeted toward men and of adopting appropriate strategies to engage men in FP conversations to reduce barriers women face in accessing FP information and methods (Potasse and Yaya, 2021).

In Kenya, there are inequities in accessing and navigating digital spaces resulting in a digital divide between those who have access to technology and data (such as phones or laptops) and those who are not able to access digital spaces due to other factors such as wealth or digital literacy (World Bank, 2019; Yousef et al., 2021). The digital divide has been exacerbated during COVID-19 (Beaunoyer et al., 2020), where studies have found that those living in peri-urban or low-income settings face difficult choices between accessing technology to stay connected to social networks remotely and meeting other daily needs, such as purchasing food (Hassan et al., 2021a). However, we found those able to access and navigate digital media spaces faced other barriers such as social stigma in accessing and sharing information on FP. Yet, despite norms and stigma in speaking about FP openly, we found that young women and their social networks were willing to find and share information on FP primarily through social media apps that they already use and were open to engaging with others to increase awareness of FP. While access to technology and the internet improves, studies highlight how advances in technology could remove other barriers to accessing FP information online, such as the ability to send voice-notes rather than texting to overcome literacy (Castle and Silva, 2019).

Online spaces present opportunities for broader diffusion of accurate information on FP from formal sources of information such as healthcare workers, as well as reducing stigma to access FP information from trusted sources (Makenzius et al., 2019). However, the ability for misinformation about FP to spread quickly in digital media channels is of great concern as research has found misinformation as one of the biggest barriers for FP uptake (Diamond-Smith et al., 2012; Ochako et al., 2015; Mwaisaka et al., 2020). While misinformation may travel along other methods of communication, such as in-person or by phone call, there is potential for misinformation to spread virally, intentionally or not, on digital media channels. Our findings indicate that some people were aware of and could identify FP misinformation online, and others sought healthcare provider's websites to minimize their contact with misinformation. However not all digital media users can recognize or fact-check suspected misinformation and has been the subject of research into methods to combat “fake-news” or misinformation online (De Beer and Matthee, 2021). Women and their key influencers described actively avoiding any sharing of misinformation online to improve informed choices on FP use in their networks. Further, we found that online spaces provided multiple means to validate information before sharing it further within networks that presents an opportunity for trusted sources to challenge misconceptions and myths about FP in online settings. While norms which sanction the use of FP may prevent conversations about FP in-person, social media groups provide online spaces where it may be more acceptable to receive and share information about FP.

This study has several limitations. While our findings provide insights into the ways in which young women and their key influencers use digital media to access and share information on FP, the findings in this paper cannot be generalized and do not represent the experiences of all young women and key influencers living in these wards. The screening process prior to the phone-based interviews also ensured that participants were comfortable to speak about FP and thus our findings do not reflect on the attitudes and norms held by participants who were not comfortable speaking about FP by phone living in peri-urban Nairobi wards. One of the inclusion criteria for this study was ownership of a smartphone and we therefore do not capture the barriers women without smartphones face. Given the challenge of collecting data physically during the COVID-19 pandemic, phone interviews were used and may have caused reluctance for participants to talk about FP or had them present what they thought were socially desirable answers. Using phone interviews however was appropriate for this study as it enabled us to reach participants despite the restrictions of movement during COVID-19. Finally, we could not ascertain whether concerns of misinformation were specifically linked to digital media or a general issue of accessing FP information.

Our findings have several implications for interventions and further research on access to FP information in this context. With a growing number of young people using digital media, FP organizations should leverage digital media channels to reach young people with youth-friendly FP content that is supported by trusted healthcare providers. Such services could complement in-person health services, or encourage young people to seek health services. While formal sources of FP information have predominantly remained physical and offline, FP programmes should seek to develop and test innovative strategies to disseminate FP information in digital spaces. Digital media research has focused on how information is disseminated through online platforms and the potential of digital information to shift behavior, where future research should focus on exploring processes through which individuals seek FP information online and their preferences of how to access trusted FP information. Further studies could focus on how social norms around sharing and accessing sensitive information online shift from online to offline settings, and test digital interventions to improve access to FP information.

## Data availability statement

The datasets generated and analysed for this study are not currently publicly available due to the sensitive nature of the qualitative data that can be linked back to individuals. They are available from the author on reasonable request.

## Ethics statement

This study was conducted in accordance with the Declaration of Helsinki. Permission to conduct this study was obtained from Strathmore University, Nairobi (ref SU-IERC0898/20) and the London School of Hygiene and Tropical Medicine (ref 22480). Written informed consent for participation was not required for this study in accordance with the national legislation and the institutional requirements. Participants provided verbal consent prior to interviews and identifying information was removed from transcripts prior to analysis.

## Author contributions

BC, KK, and AB were responsible for the conceptualization and design of the original study with contributions from AZ-A, RH, AS, CO, and KG. AS, CO, and KG were responsible for the overall supervision of the study. RH, AB, AZ-A, KG, and BC were responsible for data quality. AZ-A, AS, and KG were responsible for data analysis and interpretation. AZ-A, SS, and RH drafted the manuscript. All authors read, reviewed and approved the final manuscript.

## Funding

This was funded by TRANSFORM which is a unique joint initiative between Unilever, the UK's Foreign, Commonwealth and Development Office (FCDO) and EY.

## Conflict of interest

The authors declare that the research was conducted in the absence of any commercial or financial relationships that could be construed as a potential conflict of interest.

## Publisher's note

All claims expressed in this article are solely those of the authors and do not necessarily represent those of their affiliated organizations, or those of the publisher, the editors and the reviewers. Any product that may be evaluated in this article, or claim that may be made by its manufacturer, is not guaranteed or endorsed by the publisher.

## Footnotes

^1^Zinke-Allmang, A., Bhatia, A., Gorur, K., Hassan, R., Shipow, A., Ogolla, C., et al. (2021). *The Role of Partners, Parents and Friends in Shaping Young Women's Reproductive Choices in Peri-Urban Nairobi: A Qualitative Study*. Under review.
